# Chronic Carbonate Alkalinity Exposure Induces Dysfunction in Ovary and Testis Development in Largemouth Bass *Micropterus salmoides* by Oxidative Damage and Sex-Specific Pathways

**DOI:** 10.3390/antiox14091042

**Published:** 2025-08-23

**Authors:** Jixiang Hua, Yifan Tao, Wen Wang, Hui Sun, Taide Zhu, Siqi Lu, Bingwen Xi, Jun Qiang

**Affiliations:** 1 Wuxi Fisheries College, Nanjing Agricultural University, Wuxi 214081, China; 2 Key Laboratory of Freshwater Fisheries and Germplasm Resources Utilization, Ministry of Agriculture and Rural Affairs, Freshwater Fisheries Research Center, Chinese Academy of Fishery Sciences, Wuxi 214081, China

**Keywords:** saline-alkaline water, environmental stress, gonadal development, antioxidant, apoptosis, RNA-seq

## Abstract

Saline–alkaline water resources are globally widespread, and their rational development offers significant potential to alleviate freshwater scarcity. Saline–alkaline water aquaculture farming not only affects fish growth and survival but also impairs reproductive and developmental functions. Largemouth bass (*Micropterus salmoides*), an economically important fish, has demonstrated excellent high tolerance to such environments, in order to investigate the effects of alkaline water aquaculture environments on its growth performance, sex hormone levels, gonadal development, and molecular adaptation mechanisms. In this study, largemouth bass were chronically exposed to freshwater (0.55 mmol/L), low alkalinity (10 mmol/L), or high alkalinity (25 mmol/L) and cultured for 80 days. Alkalinity exposure more severely impacted the growth rate of females. High alkalinity significantly increased the hepatosomatic index and decreased the gonadosomatic index in both sexes; moreover, it induced oxidative stress in both sexes, evidenced by reduced superoxide dismutase (SOD), catalase (CAT), and total antioxidant capacity (TAOC) levels and elevated malondialdehyde (MDA) content. Furthermore, the levels of sex hormones Serum estradiol (E2), 11-ketotestosterone (11-KT), and testosterone were significantly reduced, accompanied by either an elevated ratio of primary oocytes and follicular atresia, or by reduced spermatogenesis. Apoptotic signals appeared in gonadal interstitial cells, with upregulated expression of genes *P53*, *Bax*, *Casp3*, and *Casp8*. Ultrastructural damage included fewer mitochondria and cristae blurring, further indicating tissue damage causing dysfunction. Transcriptome results showed that oxidative stress damage and energy metabolism imbalance caused by carbonate alkalinity were key to the delayed gonadal development, which was mainly manifested in enrichment of the ECM–receptor interaction and PI3K-Akt signaling pathways in females exposed to low alkalinity, and the GnRH secretion and chemokine signaling pathways in males. Glycosphingolipid biosynthesis and Ferroptosis pathway were enriched in females exposed to high alkalinity, and the Cortisol synthesis and secretion pathway were enriched in males. Overall, high-alkalinity exposure significantly delayed gonadal development in both sexes of largemouth bass, leading to reproductive impairment.

## 1. Introduction

China leads global aquaculture production, contributing over 60% of the world’s total output, with freshwater systems accounting for 58.8% of this share [1]. Despite possessing substantial water resources nationally, the country’s per capita freshwater availability is only 1827 m^3^, approximately one-quarter of the global average [2]. Increasing demands for industrial and domestic water use are intensifying pressure on freshwater supplies, consequently constraining the potential for freshwater aquaculture expansion. Concurrently, rapid development of the aquaculture sector and rising consumer living standards are driving increased demand for high-quality aquatic products. The strategic utilization of underused saline–alkaline water resources thus presents a significant approach for alleviating freshwater scarcity. China has extensive areas of saline–alkaline land and water. The total area of saline–alkaline land exceeds 99 million hectares (ha), with low-lying saline–alkaline water bodies covering ~46 million ha; both figures show an increasing trend annually [3,4].

Characterized by high carbonate alkalinity, elevated pH, a complex hydrochemical composition, and an imbalanced ion composition [5,6], saline–alkaline waters are generally unsuitable for most aquatic species. Nevertheless, certain fish species have adapted to survive in highly saline and alkaline lakes, such as China’s Lake Qinghai and Dali Lake. These fish respond to osmotic pressure changes through adaptive changes in tissue structure such as gills, kidneys, and intestines, and have evolved efficient ammonia nitrogen excretion mechanisms to reduce ammonia toxicity, these unique physiological mechanisms allow them to adapt to extreme environments [7,8]. Understanding the growth and reproduction of these resilient species is therefore a prerequisite for developing saline–alkaline tolerant aquaculture strains.

High levels of carbonate alkalinity act as an abiotic stressor for aquatic organisms by influencing water pH and ionic equilibrium, thereby disrupting osmoregulation, acid–base balance, and ammonia nitrogen metabolism [9]. Research indicates that the gill, kidney, and intestine are the primary organs involved in the response of aquatic animals to carbonate–alkaline stress [10,11]. Under alkaline stress, freshwater fishes such as the spotted scat *Scatophagus argus* and crucian carp *Carassius gibelio* exhibit significant alterations in gill morphology, including gill filament swelling and curling, chloride cell hypertrophy, and hyperplasia and detachment of epithelial cells [12,13]. The Lahontan cutthroat trout *Oncorhynchus clarkii henshawi*, uniquely adapted to long-term residence in saline–alkaline waters, displays kidney tissue degeneration and glomerular atrophy [14]. Conversely, Nile tilapia *Oreochromis niloticus* and Amur minnow *Phoxinus lagowskii* respond to carbonate–alkaline exposure with increased intestinal villus height, elevated goblet cell numbers, and concomitant epithelial cell sloughing [11,15,16]. These structural changes in organs often represent adaptations to maintain ammonia nitrogen metabolism, the acid–base balance, and osmotic equilibrium. An efficient ammonia excretion system is fundamental for maintaining physiological homeostasis in aquatic organisms. In high-alkalinity environments, the decomposition of carbonates elevates water pH and intensifies hydrogen ion (H^+^) efflux, which may hinder ammonia excretion; this can lead to toxic ammonia accumulation or ammonia poisoning [17]. For instance, high-alkalinity exposure significantly suppresses ammonia excretion in the Chinese mitten crab *Eriocheir sinensis*, inducing oxidative stress and apoptosis, culminating in mortality [18]. In fish, elevated pH in the environment can create a substantial acid–base gradient inside and outside the fish’s body. As the primary interface with the aquatic environment, the gills expel substantial H^+^ during acid–base regulation, exacerbating CO_2_ loss and potentially triggering respiratory alkalosis [19]. Osmoregulation is intrinsically linked to ammonia metabolism and the acid–base balance. To counter hyperosmotic conditions, the gills facilitate transmembrane transport of ions, molecules, and water to regulate internal osmotic pressure [20]. Thus, excessive carbonate alkalinity exposure can disrupt the dynamic equilibrium among these three regulatory processes, ultimately impairing survival, development, and reproduction in aquatic animals.

The reproductive system of aquatic organisms is extremely sensitive to environmental stresses [21]. Environmental factors like dissolved oxygen, temperature, and pH significantly influence the endocrine system in fish, particularly the hypothalamic–pituitary–gonadal (HPG) axis which regulates reproduction. Oxidative damage to gonad tissues interferes with gametogenesis and disrupts the expression of key sex-regulated genes; consequently, the reproductive system can be harmed, lowering reproductive performance [22,23,24]. For example, chronic exposure of Nile tilapia and yellow catfish *Pelteobagrus fulvidraco* to low dissolved oxygen (2 mg/L) resulted in a significant decrease in serum estrogen levels, an increase in the number of follicular atresia, and a concomitant increase in apoptosis in gonadal cells, leading to reproductive impairment [15,21]. Similarly, steroid hormones are crucial for the reproductive process in chordates. Cholesterol undergoes a series of enzymatic reactions to produce different steroid hormones, and the well-known estrogen estradiol (E2), vitellogenin (VTG), follicle-stimulating hormone (FSH), and androgens like testosterone (T), and 11-ketotestosterone (11-KT) are involved in gonadal differentiation and maturation, which, together with the HPG axis, affect the reproductive system, and synergistically affect the reproductive system [25]. Carbonate alkalinity, a crucial factor in saline–alkaline water, was found to retard follicular development in the ridgetail white shrimp *Exopalaemon carinicauda* in response to a high alkalinity level of 8 mmol/L [26]. In another comparison of the reproductive performance of that species in response to prolonged exposure to alkaline stress, the parent shrimp was unable to hold eggs at alkalinity levels of >8 mmol/L [27]. In medaka fish *Oryzias latipes*, the hatching rate of fertilized eggs was also inhibited at alkalinity levels of >5.3 mmol/L [4]. To date, studies on the response of aquatic organisms to carbonate–alkaline stress have focused mostly on the mechanisms of alkalinity tolerance and immune responses, whereas little research has been conducted on the adaptive mechanisms regulating gonadal development and reproduction.

Largemouth bass *Micropterus salmoides*, an economically significant fish species in China, prized for its palatable flesh and lack of intermuscular spines, is commercially farmed in most provinces. Previous studies have shown that largemouth bass can tolerate a salinity and alkalinity up to 11‰ and 25 mmol/L, respectively, demonstrating an excellent tolerance to saline–alkaline conditions [28,29]. Saline–alkaline aquaculture is currently carried out in Shandong, Henan, and Shaanxi provinces in China. However, the regulatory mechanism of the reproductive system of largemouth bass in response to carbonate alkalinity stress has not yet been clarified, and carbonate alkalinity may impair reproductive function through the following mechanisms: (i) increased levels of oxidative stress in the gonads, which inhibits gametogenesis; (ii) dysregulation of sex hormone levels, which delays gonadal development; (iii) activation of the sex-specific apoptosis pathway, which leads to damage to germ cells leading to germ cell damage. Therefore, this study compared the growth performance, gonadal histology and microstructure, oxidative stress, sex hormone levels, apoptosis, and transcriptome analysis between male and female largemouth bass under long-term exposure to different levels of carbonate alkalinity, to comprehensively determine the impact of alkaline water on their reproductive performance.

## 2. Materials and Methods

### 2.1. Experimental Fish and Carbonate Alkalinity Exposure

The experiment used fish from the F_2_ generation of the foundation population of largemouth bass introduced from the United States, which in 2020 were being reared by the Freshwater Fisheries Research Center (FFRC) of the Chinese Academy of Fisheries Sciences. Six hundred largemouth bass with good feeding, similar size, and average weight of (18.19 ± 0.14 g) were selected and temporarily cultured in 20 recirculating water culture buckets (400 L) to adapt to the culture environment. The fish were then temporarily cultured for 2 weeks, during which time the water temperature was maintained at 26 ± 1 °C, dissolved oxygen at ≥7.5 mg/L, and total ammonia nitrogen and nitrite at ≤0.03 mg/L, under a photoperiod of 14L:10D. The fish were fed a commercial feed (≥47% crude protein, and ≥3% crude fat) twice a day, at 8:00 and 16:30. Following acclimatization, an 80-day carbonate–alkaline exposure experiment was initiated.

The concentration of carbonate used in this study was based on the lethal concentration (96h-LC_50_) and the alkalinity of idle saline–alkaline water [29]. The experimental set up comprised a freshwater control group (0.55 mmol/L [CA0]), a low-alkalinity exposure group (10 mmol/L [CA10]), and a high-alkalinity exposure group (25 mmol/L [CA25]). Each group consisted of three replicates of 30 fish each, without distinguishing between males and females, yielding a total of 270 experimental fish, which were randomly distributed among nine recirculating water aquaculture buckets (400 L). The exposure concentration of the low- and high-alkalinity groups was configured using NaHCO_3_ (analytically pure) according to the volume of the culture water, and the alkalinity was increased by 5 mmol/L increments per day to reach the final experimental alkalinities.

Once the exposure experiment was initiated, the number of deaths of fish in each group was recorded daily during the 80-day culture period. During the experiment, the alkalinity of the water was tested daily by acid–base titration. Hydrochloric acid-methyl orange was used as an indicator to maintain the given alkalinity. During the experimental period, the environmental conditions required for gonadal development were consistent with the reproductive and developmental needs of largemouth bass. The water temperature was maintained at 26 ± 1 °C, the dissolved oxygen was ≥7.5 mg/L, the total ammonia nitrogen and nitrite were ≤0.03 mg/L, and the photoperiod was 14L:10D. Water was replaced every three days, and the alkaline water was reconstituted. Residual food and feces were promptly removed by siphoning to maintain clean water.

### 2.2. Sample Collection

At the end of the exposure experiment, the fish were fasted for 24 h before the final body weight of 30 experimental fish was determined for each group (15 females and 15 males, dissected to confirm the sex). To calculate the hepatosomatic index (HSI) and gonadosomatic index (GSI), the fish were individually weighed after anesthesia with 100 mg/L of MS-222 [30]. This was followed by rapid dissection on crushed ice to remove the overall visceral mass, and to isolate the liver and gonadal tissues. Subsequently, gonadal tissues from two females and two males were randomly collected from each of the three replicate tanks in each group; the gonads were evenly divided into three parts using scissors, washed with 1% saline, and then two of the parts were placed in 4% paraformaldehyde solution and fixed at 4 °C for 24 h for later histological observation and apoptotic cell death analysis. The other part of the gonad was trimmed to the size of 2 mm × 2 mm and then placed in 2.5% glutaraldehyde solution, fixed at room temperature for 2 h, and then stored at 4 °C until use for transmission electron microscopy (TEM) analysis. Another 0.5 g of gonadal tissue was dissected from three females and three males randomly collected from each breeding bucket, with nine experimental fish collected from each group; these tissue samples were placed in liquid nitrogen for quick-freezing, stored at −80 °C, and finally the samples were mixed, by sex, for transcriptome sequencing of males and females. The number of samples collected is shown in Table S1.

The specific growth rate (SGR), HSI and GSI were calculated as follows:

$$SGR\;\left(\%\right)=[(\ln W_{t}-\ln W_{0})/t]\times 100$$

$$HSI\;(\%)={(L}_{w}/W_{t})$$

$$GSI\;(\%)={(G}_{w}/W_{t})\times 100$$

where $W_{t}$ and $W_{0}$ are the final and initial body weights, respectively, and L_*w*_ and G_*w*_ are the liver and gonad weights, respectively.

### 2.3. Antioxidant Indicators and Sex Hormone Measurements

Three females and three males were randomly selected from each of the three parallel culture buckets of groups CA0, CA10, and CA25, that is, nine males and nine females for each experimental group, respectively. Then, 1.5 mL of blood was withdrawn from the tail vein and left to stand for 2 h. The samples were centrifuged at 5000 r/min for 15 min at 4 °C, and the separated serums were stored at −20 °C for reserve. Serum antioxidant enzymes catalase (CAT, BPC005), superoxide dismutase (SOD, BPC004), total antioxidant capacity (TAOC, BPC011), and malondialdehyde (MDA, BPC001) were measured using commercial kits from Shanghai Haoben Biological Co., Ltd. (Shanghai, China). The serum levels of estradiol (E2) and vitellogenin (VTG) in females, and the serum levels of 11-ketotestosterone (11-KT) and testosterone (T) in males were determined by enzyme-linked immunosorbent assay (ELISA). The detailed assay procedures were performed according to the kit instructions.

### 2.4. HE Staining

Paraffin-embedded sections of the fixed gonadal tissue samples were prepared for histological observation. Briefly, the HE staining process consisted of dehydrating the gonadal tissues step by step in different concentrations of ethanol solution (70%→100%), clearing the tissues in xylene/ethanol (1:1) solution, dipping the waxes twice, then placing them into melted paraffin wax for embedding, and finally making 4 μm-thick sections after solidification. Each tissue sample was sectioned three times, and the sections were observed using an optical microscope (CX22LED, Olympus, Tokyo, Japan), with three fields of view captured per section. The degrees of ovarian and spermathecal development were measured using Image J (1.54d) software. The ratio of the occurrence of primary growth oocytes and follicular atresia was counted in each field of view. The ratio of spermatozoa to the seminiferous tubules (%) was calculated as follows: [area of spermatozoa/area of seminiferous tubules] × 100.

### 2.5. TUNEL Staining

To detect apoptosis, paraffin sections of the gonadal tissue samples were prepared and subjected to TUNEL staining. First, the paraffin sections were deparaffinized by xylene, hydrated with different concentrations of ethanol solution (100%→70%), washed with PBS, and incubated with proteinase K (20 μg/mL) for 15–30 min at room temperature for permeabilization. Next, the sections were evenly covered with TUNEL reaction solution (a mixture of TdT and DIG-dUTP) and incubated in a humidified box at 37 °C while protected from light for 1 h. After washing with PBS three times, the sections were re-stained with a drop of DAPI staining solution (0.5 μg/mL) for 5 min. After shaking dry to seal the sections, the sections were observed using a fluorescence microscope (E100, DS-U3, Nikon, Tokyo, Japan), and then scanned using SlideViewer (3DHISTECH 3.0.5) slide-viewing software, with nine fields of view scanned in each group. Finally, the apoptosis rates of ‘positive cells’ in the ovaries and testes were counted using Image-Pro Plus 6.0 image-processing software. Positive apoptotic cells were determined by emitting green-fluorescent signals, and the apoptosis rate was calculated as the ratio of the number of apoptotic cells to the total number of cells.

### 2.6. TEM Analysis

Gonadal tissues were fixed in 2.5% glutaraldehyde for 24 h, rinsed three times (15 min) using 0.1 M of PBS, then transferred to 1% osmium tetroxide solution, and fixed for another 2 h at room temperature and protected from light, and finally rinsed three times again in 0.1 M of PBS. The gonadal tissues were dehydrated in a series of ethanol solutions (50%, 70%, 90%, and 100%), step by step for 15 min, and then immersed for 2 h in a solution of epoxy resin mixed with an equal proportion of acetone, then immersed in pure resin overnight, poured into embedding cassettes, and polymerized for 48 h at 60 °C. The solidified resin was trimmed, and 50–70 nm ultrathin sections were cut using an ultrathin sectioning machine and then mounted on copper mesh grids covered with a Formvar film. Finally, the sections were stained with 2% uranyl acetate while protected from light, for 15 min, washed with double-distilled water and stained with lead citrate for 5 min, and dried. These sections were observed using a Hitachi HT7800 transmission electron microscope (Hitachi High-Tech, Hitachinaka, Tokyo, Japan) at 80–120 kV, each tissue sample was sectioned four times; two fields of view were captured for each section, and images were captured for analysis.

### 2.7. Transcriptome Sequencing

#### 2.7.1. RNA Extraction, Library Construction, and Sequencing

Six treatment groups were set up: F0 (control females), M0 (control males), F10 (low-alkalinity group females), M10 (low-alkalinity group males), F25 (high-alkalinity group females), and M25 (high-alkalinity group males); each treatment group had 3 parallels, and 3 experimental fish were randomly selected from each parallel, and 9 experimental fish were collected from each treatment group, for a total of 54 samples. Total RNA was extracted by the Trizol method [31]; the integrity of RNA samples was assessed with 1% agarose gel electrophoresis, after using DNase I enzyme to remove DNA contamination, and measured with a NanoPhotometer N50 spectrophotometer (Implen, Munich, Germany). Three RNA samples were randomly selected from each treatment group; 1.0 μg aspirated aliquots from three biological replicates in each group were mixed to form a sample pool, for the construction of cDNA libraries.

Library construction and high-throughput sequencing were performed with the assistance of Gidi Biotechnology Co., Ltd. (Guangzhou, China). The enriched mRNA was isolated and interrupted, and the double-stranded cDNA was synthesized from the reverse transcriptase system, which was purified, end-repaired, A-tailed, and connected to the sequencing junction, and then PCR-amplified and purified to obtain the final library. The library was subjected to 2 × 150 bp double-end sequencing on the Illumina HiSeq 4000 platform (Illumina, San Diego, CA, USA).

#### 2.7.2. Differential Gene Expression Screening and Functional Enrichment Analysis

The raw sequencing data were quality-controlled by removing the low-quality data, and the quality-controlled clean reads were aligned with the reference genome of largemouth bass (available online: https://www.ncbi.nlm.nih.gov/datasets/genome/GCF_014851395.1 (accessed on 10 December 2023)) using HISAT2 2.2.1 software. Based on the results of the comparison with HISAT2, the expression of genes was quantified for each sample using RSEM (1.3.3) software after reconstructing the transcripts using StringTie (v2.2.1). DESeq (1.48.1) software was used to perform differential gene expression analysis, with significant differentially expressed genes (DEGs) filtered based on the criteria of |log2FoldChange| > 1.5, and *p* < 0.05. The DEGs were mapped to the GO Ontology database and KEGG pathway database to understand their functional roles and involvement in biological pathways, and the number of DEGs was calculated. Lastly, the hypergeometric distribution method was used, when *p* < 0.05 was considered to be significantly enriched for DEGs in the GO term and KEGG pathway.

#### 2.7.3. Protein–Protein Interaction (PPI) Network Construction and Hub Gene Screening

For this, three comparison groups were set up with females: F0 vs. F10, F0 vs. F25, and F10 vs. F25; likewise, three comparison groups were set up with males: M0 vs. M10, M0 vs. M25, and M10 vs. M25. KEGG-enriched pathways shared in two or more comparison groups were selected for males and females. All the genes in the pathway were imported into the STRING database (https://cn.string-db.org) to form a gene set; the generated gene set network data were used to construct an interoperability network using Cytoscape 3.10.0 software, and the hub genes were screened by the MCC algorithm based on the Cyto-Hubba plug-in.

#### 2.7.4. Gene Set Enrichment Analysis

All genes in the pathways of focus in the female–male comparison group were subjected to GSEA using the KEGG pathway. Briefly, GSEA 2.2.4 software was used to rank the expression of all genes in the pathway, score the pathway, calculate the enrichment score (ES), calculate the *p*-value using the permutation test, and correct the normalized enrichment score (NES) value after normalization of the ES value by multiple testing to obtain the FDR value. The pathway gene set was considered significant when |NES| > 0.85, the NOM *p*-value was <0.05, and the FDR *q*-value was <0.25.

### 2.8. RT-qPCR Analysis

The genes *P53*, *Bax*, *Casp3* and *Casp8*, which are related to apoptosis, were selected for further validation of TUNEL-positive apoptotic signaling. To verify the reliability of transcriptional level changes in the gonadal tissues of males and females in the different alkalinity exposure groups, three genes were selected in each of the three comparison groups of males and females, and RT-qPCR was used to verify the gene expression patterns. Following the validation method of Wang et al. [32] with β-actin as the internal reference gene, the primers used were designed based on primers used by Pi et al. [33] and Chen et al. [22] from the genome of largemouth bass, using Primer 6.0 software; three independent biological replicates were used in each group, and each replicate was validated three times. Supplementary Materials Table S2 lists information on the primers, calculated using the 2^−ΔΔCt^ method for comparing the relative expression level of the target gene [34].

### 2.9. Data Analysis

The normality and homogeneity of variance of the experimental data were checked using SPSS 22.0 (IBM Corp, Ammonk, NY, USA). Then, the degree of significance of the difference between the groups was analyzed using Duncan’s multiple comparisons in one-way ANOVA; *p*-value < 0.05 was considered significant. All data are expressed as mean ± standard deviation (SD). Correlation analysis and clustering heatmaps were performed using Lianchuan Bio Cloud Analytics (available online: https://www.omicstudio.cn/home (accessed on 10 December 2024)) and GraphPad Prism 9.0.

## 3. Results

### 3.1. Comparison of Growth Performance

**Figure 1:**
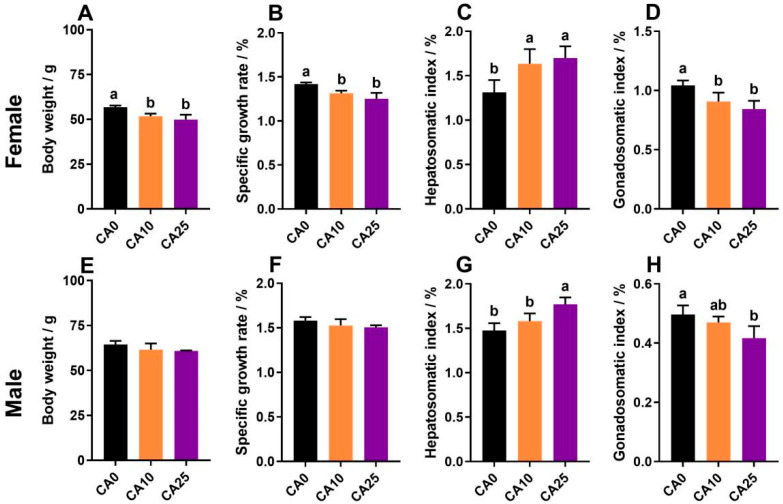
Effects of carbonate alkalinity exposure on growth performance of largemouth bass. (**A**–**D**) Final body weight, specific growth rate, hepatosomatic index, and gonadosomatic index of female largemouth bass; (**E**–**H**) Final body weight, specific growth rate, hepatosomatic index, and gonadosomatic index of male largemouth bass. Differences between groups are indicated by different lowercase letters (*p* < 0.05).

After 80 days of exposure, the body weight and SGR of females in the groups CA10 and CA25 were significantly lower than those in the control; the HSI was significantly higher, and the GSI was significantly lower than that of the controls, and there was no significant difference in growth performance between the two treatment groups (Figure 1A–D). The body weight and SGR of males in groups CA10 and CA25 did not differ significantly from those of the control group, whereas the HSI tended to be higher and the GSI lower for those groups as compared with the controls; both the HSI and GSI of group CA25 differed significantly from the controls (Figure 1E–H).

### 3.2. Antioxidant Indices and Variation in Sex Hormone Levels

**Figure 2:**
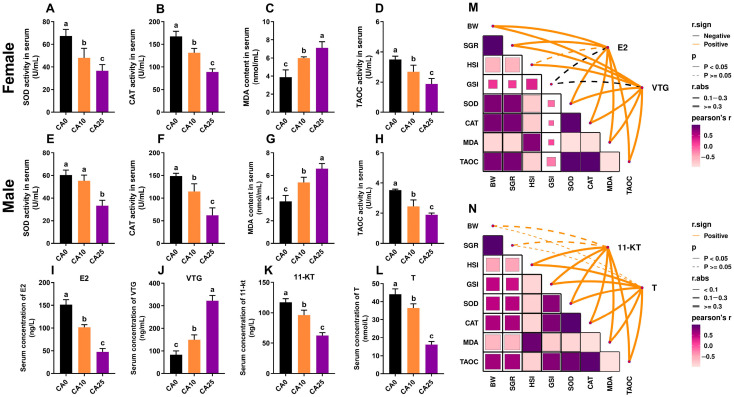
Effects of carbonate alkalinity exposure on changes in antioxidant indices and sex hormone levels in largemouth bass. (**A**–**D**) Serum antioxidant indices of superoxide dismutase (SOD), catalase (CAT), malondialdehyde (MDA), and total antioxidant capacity (TAOC) in females; (**E**–**H**) Serum antioxidant indices of SOD, CAT, MDA, and TAOC in males; (**I**,**J**) Serum estradiol (E2) and vitellogenin (VTG) levels in females; (**K**,**L**) Serum 11-ketotestosterone (11-KT) and testosterone (T) levels in males; (**M**,**N**) Correlation network heatmap representing the relationship of sex hormones with growth performance and antioxidant indexes. Network correlations were analyzed by Mantel’s test; heatmap correlations were analyzed by Spearman’s test. Differences between groups are indicated by different lowercase letters (*p* < 0.05).

In female largemouth bass, after 80 days of exposure the change in the serum antioxidant indices of SOD, CAT, and TAOC showed a decreasing trend, while the MDA content showed an increasing trend, and there were significant differences among the three groups (Figure 2A–D). After 80 days of exposure, males showed the same trend of changes in the antioxidant indices as females, but with no significant difference in the level of SOD activity between group CA10 and the control (Figure 2E–H). At higher alkalinities, the serum E2 level in females decreased significantly, and the VTG levels increased significantly (Figure 2I,J), whereas in males the serum 11-KT and T levels both decreased significantly (Figure 2K,L). These changes in sex hormone levels were more significant for females.

Correlations between the sex hormone levels and growth performance and antioxidant indices were further analyzed for males and females. In females, the E2 level was not correlated with the HSI and GSI, and the VTG level was not correlated with the GSI, but the E2 and VTG levels were positively correlated with the rest of the indices (*p* < 0.05). In males, the 11-KT and T levels were not significantly correlated with BW and the SRG (*p* ≥ 0.05) but were positively correlated with the rest of the indices (*p* < 0.05). In addition, strong positive correlations between growth performance and the antioxidant indices were observed in both females and males (Figure 2M,N).

### 3.3. Gonadal Histology and Apoptosis Analysis

**Figure 3:**
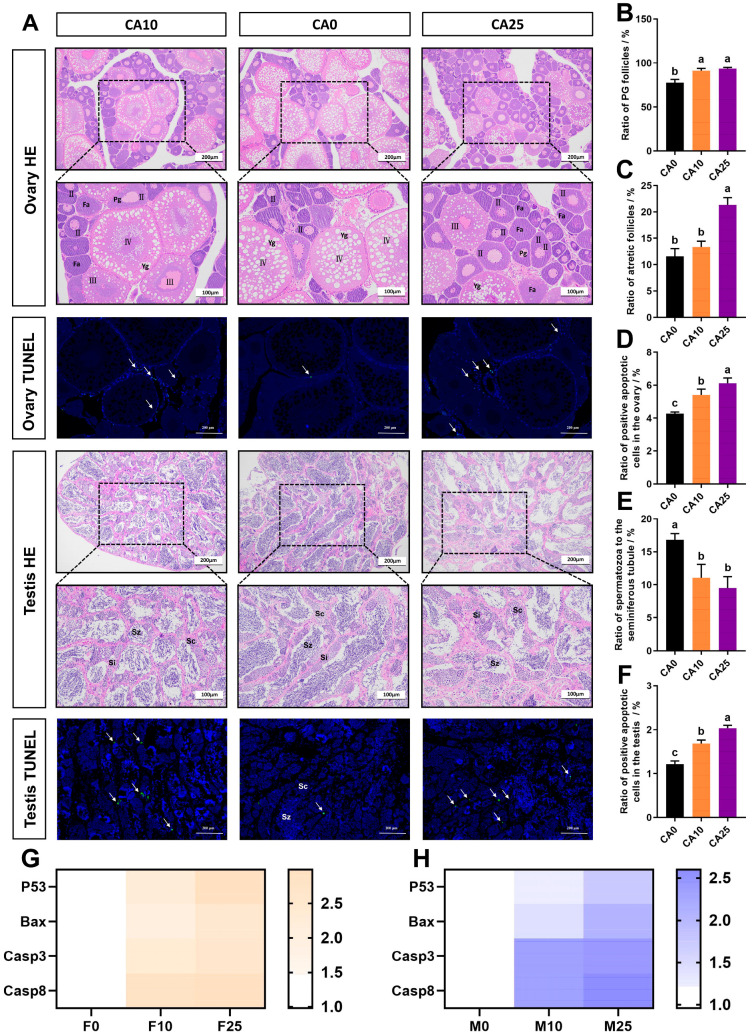
(**A**) Observations of hematoxylin–eosin (HE)-staining sections (×100 and ×200) and terminal deoxynucleotidyl transferase-mediated dUTP nick end labeling (TUNEL)-staining positive apoptotic signals (×100) of largemouth bass ovaries and testes under carbonate alkalinity exposure; (**B**) Statistics on the proportion of primary growth oocytes in the ovaries after exposure; (**C**) Statistics on the proportion of atretic follicles in the ovaries after exposure; (**D**) Statistics on TUNEL-positive apoptotic signals in the ovaries after exposure; (**E**) Statistics of the proportion of spermatogenesis in the seminiferous tubules after exposure; (**F**) Statistics of TUNEL-positive apoptotic signaling in the testes after exposure; (**G**) Changes in the expression of apoptosis-related genes in the ovary after exposure; (**H**) Changes in the expression of apoptosis-related genes in the testes after exposure. Pg: Primary growth oocyte; Yg: Yolk granules; Fa: Follicle atretic; Sc: Secondary spermatocyte; Si: Spermatid; Sz: Spermatozoon; II, III, and IV represent oocytes of different periods; Arrows mark positive apoptotic cells. Differences between groups are indicated by different lowercase letters (*p* < 0.05).

Examples of stained gonadal tissue sections of males and females after exposure to carbonate alkalinity are shown in Figure 3A. In the control group, in the early stage of development of the ovary, up to period IV, the ovaries are filled with oocytes in their late developmental stage, characterized by a large accumulation of yolk granules; also obvious are the thickening of radial bands, the nuclei undergoing polarization, the transition to the maturity state, and the boundaries of the double layer of follicular membranes. By comparison, ovarian development was inhibited in group CA10 females; the tissue sections show the oocytes are predominant in periods II and III, but few in period IV, with their nuclei not yet fully contracted and polarized, and follicular atresia was beginning to occur. In group CA25 females, ovarian development was significantly inhibited, with oocytes only found in periods II and III, and with many follicles undergoing atresia.

Analysis of stained gonadal tissues revealed the primary growth oocyte (PG) and follicle atretic (FA). Their incidence in ovaries gradually increased under exposure to a higher alkalinity; PG and FA were as high as 93.62% and 21.33%, respectively, in group CA25 females, which was significantly higher than in control females (Figure 3B,C). TUNEL staining of ovarian tissue showed that positive apoptotic cells appeared more often in the mesenchymal cells surrounding the oocytes, and the rates of positive apoptotic cells in the ovaries of groups CA10 and CA25 were 5.41% and 6.11%, respectively, which were significantly higher than in the control group (4.27%) (Figure 3D).

Staining showed that the testis of control fish developed gradually to stage IV, and the seminiferous lobules were filled with a large number of secondary spermatocytes, spermatids, and spermatozoa, and the spermatozoa were closely packed together in the lumen of the seminiferous lobules. In contrast, the testes of males in the exposure groups were significantly affected by alkaline water. Group CA10 males showed normal rows of seminiferous lobules, but the rows of spermatozoa in the lumen became sparse, with the presence of some large blank lumens, the thickening of interstitial tissues, and a significant decrease in spermatocytes and spermatozoa. In group CA25 males the testis was severely damaged, the arrangement of seminiferous lobules was disorganized, few spermatozoa existed in the tubular lumen, the blank area in the tubular lumen gradually increased, the volume of interstitial tissues increased, and the number of secondary spermatogonia and spermatocytes also decreased. Further statistics on the percentage of spermatozoa in the seminiferous tubules were 11.05% and 9.52% in the CA10 and CA25 groups, respectively, which were significantly lower than that of the control group (16.85%) (Figure 3E). TUNEL staining showed that the apoptosis rate of testicular positive cells increased with exposure to a higher alkalinity, at 1.68% and 2.03% in groups CA10 and CA25, respectively, both significantly higher than in the control (1.21%) (Figure 3F).

The RT-qPCR results proved that the expression of apoptosis-related genes *P53*, *Bax*, *Casp3*, and *Casp8* were upregulated in both ovary and testis following exposure to alkaline water, as compared with the control fish, but the gene expression levels were lower in testes than in ovaries, which aligned with the results of TUNEL staining (Figure 3G,H).

### 3.4. Electron Microscopy of Gonadal Tissues After Carbonate Alkalinity Exposure

Ultrastructural changes in the ovaries and testes of largemouth bass were characterized by electron microscopy after exposure to alkaline water. Overall, the exposure caused structural damage to both the ovaries and testes, with the damage being more pronounced at higher alkalinities.

**Figure 4:**
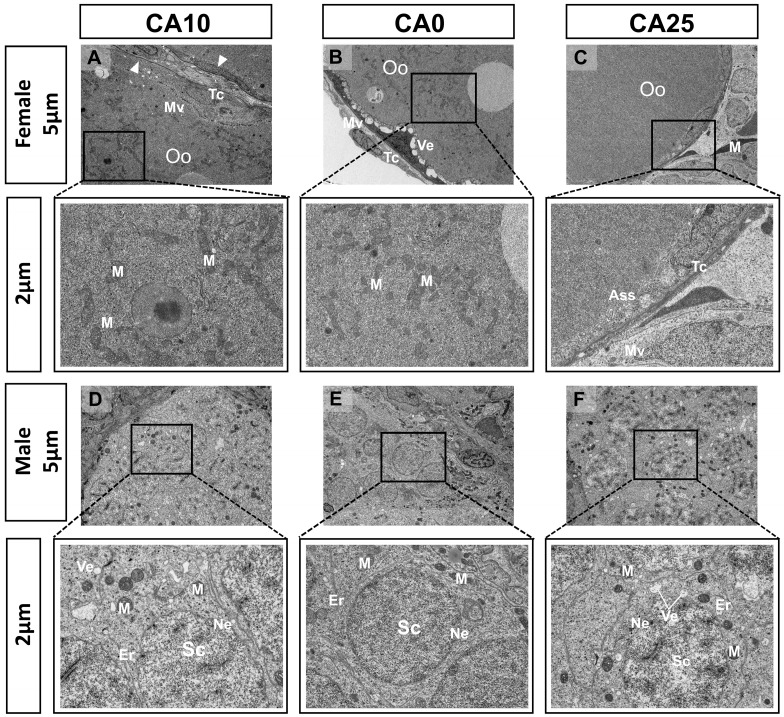
Ultrastructural observations of ovary and testis tissues after carbonate alkalinity exposure in largemouth bass (×2.5k and ×7k). (**A**) Females from group CA10; (**B**) Control females; (**C**) Females from group CA25; (**D**) Males from group CA10; (**E**) Control males; (**F**) Males from group CA25. M: Mitochondria; Oo: Oocyte; Mv: Microvilli; Tc: Theca cell; Ass: Autophagolysosome; Ve: Vesicle; Ne: Nucleus; Er: Endoplasmic reticulum; Sc: Secondary spermatocyte; White arrows mark blurred follicular membrane boundaries.

The oocytes of control group females displayed a regular structure, with intact nuclear membranes without indentations, clear boundaries of the double-layered follicular membranes, neatly arranged cortical vesicles at the outer edge of the ooplasm, neatly arranged microvilli, and mitochondria that were mostly elongated and dumbbell-shaped and possessed abundant cristae (Figure 4B). In group CA10 females, the outer follicular membrane of oocytes had blurred boundaries, the membrane cells were elongated, the microvilli were irregularly arranged, the number of mitochondria was reduced and appeared swollen, and part of the intramembranous matrix was ruptured (Figure 4A). In group CA25 females, the outer follicular membrane of oocytes was thinned, the morphology of membrane cells was distorted, the interstitium was partially edematous, the intracellular organelles of the cells were reduced, the number of microvilli was reduced, the number of lysosomes was increased, and only a small number of mitochondria were observed in the cells (Figure 4C).

In males, the secondary spermatocytes in the control group were neatly arranged, the cell membrane structure was intact, the endoplasmic reticulum was not deformed, and the mitochondria appeared as regular and elongated dumbbell shapes, with well-defined cristae (Figure 4E). In group CA10, the secondary spermatocytes were irregularly arranged, the cell membrane boundaries were blurred and concave, the number of endoplasmic reticulum was reduced and accompanied by the appearance of vesicles, there were fewer mitochondria, and they were in the form of a detached ellipsoid or spherical shape, and the cristae were blurred in their fracture (Figure 4D). In group CA25, the spermatocytes were more severely damaged, with curved and deformed cell membranes, an increased number of vesicles, drastically reduced numbers of mitochondria that were arranged in a fragmented manner to form oval and spherical shapes, cristae clustered together, disrupted endoplasmic reticulum structures, and sparse cytoplasm (Figure 4F).

### 3.5. Transcriptome Sequencing Analysis

The mRNA expression changes in ovary and testis tissues under carbonate alkalinity exposure were analyzed by transcriptome sequencing. The ovary and testis cDNA libraries were obtained with 50,161,678 raw reads and 426,164,148 raw reads, respectively, and the data were filtered to show that the percentage of clean reads was greater than 99%, with an average Q20 greater than 98%, Q30 greater than 95%, and GC percentage greater than 49% (Table S3). These results indicate the sequencing quality was high.

### 3.6. Identification and Functional Enrichment Analysis of DEGs in Ovary

**Figure 5:**
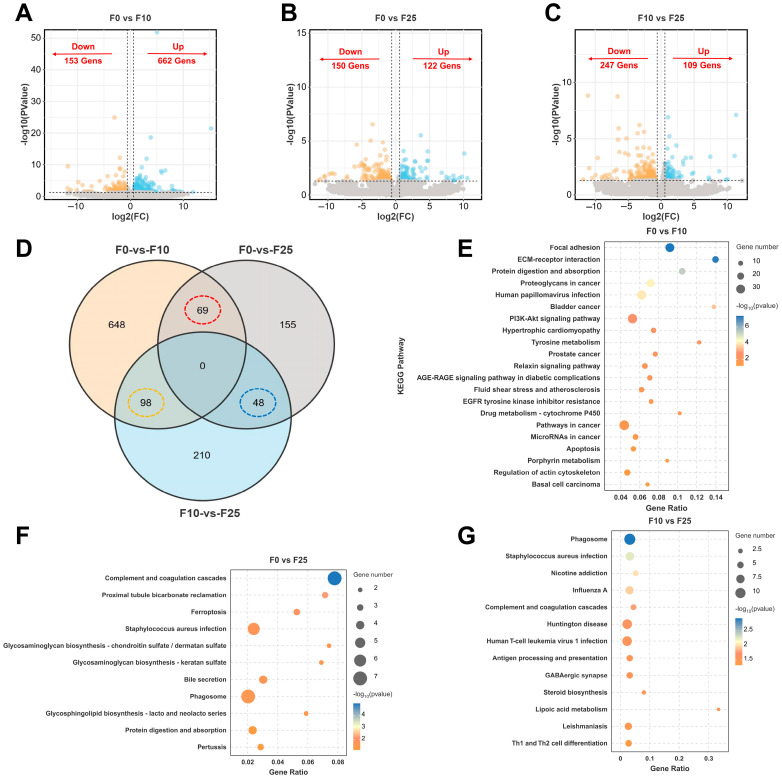
Transcriptome analysis of largemouth bass ovaries after carbonate alkalinity exposure. (**A**–**C**) Volcano plot statistics of up- and downregulated differentially expressed genes (DEGs) in the three comparison groups of F0 vs. F10, F0 vs. F25, and F10 vs. F25; (**D**) Wayne plot statistics of differences among the three comparison groups, with common DEGs circled in red, yellow, and blue, respectively. (**E**–**G**) Kyoto Encyclopedia of Genes and Genomes (KEGG) pathway statistics of DEGs obtaining significant enrichment (*p* < 0.05) in the three comparison groups F0 vs. F10, F0 vs. F25, and F10 vs. F25.

For the three comparative groups of females (i.e., F0 vs. F10, F0 vs. F25, and F10 vs. F25), a total of 815 DEGs (153 upregulated, 662 downregulated), 272 DEGs (150 upregulated, 122 downregulated), and 356 DEGs (247 upregulated, 109 downregulated) were screened, respectively (Figure 5A–C). The expression of DEGs was upregulated in F0 vs. F10, but more were downregulated in F0 vs. F25. Differential Wayne plots were drawn to visualize the overlap of DEGs in the three comparison groups, as shown in Figure 5D. No co-DEGs were identified in the three groups, but 69, 98, and 48 co-DEGs were identified in F0 vs. F10/F0 vs. F25, F0 vs. F10/F10 vs. F25, and F0 vs. F25/F10 vs. F25, respectively. Therefore, a clustered heatmap of the expression of co-DEGs was further generated. All showed different expression cluster patterns in relation to different concentrations of alkalinity (Figure S1A–D). Next, the top 15 GO terms in each of the three comparison groups were counted. The DEGs were more enriched in GO terms related to ion transport, such as metal-ion binding and cation binding, in the F0 vs. F10 group; in GO terms related to immune regulation, such as humoral immune response and positive regulation, in the F0 vs. F25 group; and more enriched in biological process (BP)-related GO terms, such as lipid metabolic process, in the F10 vs. F25 group (Figure S2A).

Focusing on the KEGG enrichment results of DEGs in the ovary under carbonate alkalinity exposure to screen potential signaling pathways and candidate genes affecting ovarian function, DEGs were significantly enriched in 21, 11, and 13 signaling pathways in the comparison groups F0 vs. F10, F0 vs. F25, and F10 vs. F25, respectively (*p* < 0.05). Pathways related to signaling and endocrine metabolism were significantly enriched in the F0 vs. F10 group, such as Focal adhesion, ECM–receptor interaction, PI3K-Akt signaling pathway, and Relaxin signaling pathway (Figure 5E); in the F0 vs. F25 group, pathways related to glycan biosynthesis and metabolism and cellular processes were significantly enriched, such as glycosphingolipid biosynthesis—lacto- and neolacto-series and Ferroptosis (Figure 5F); in the F10 vs. F25 group, enriched pathways related to the immune system and metabolism; in the F10 vs. F25 group, enriched pathways related to the immune system and metabolism, such as Steroid biosynthesis and Lipoic acid metabolism (Figure 5G).

**Figure 6:**
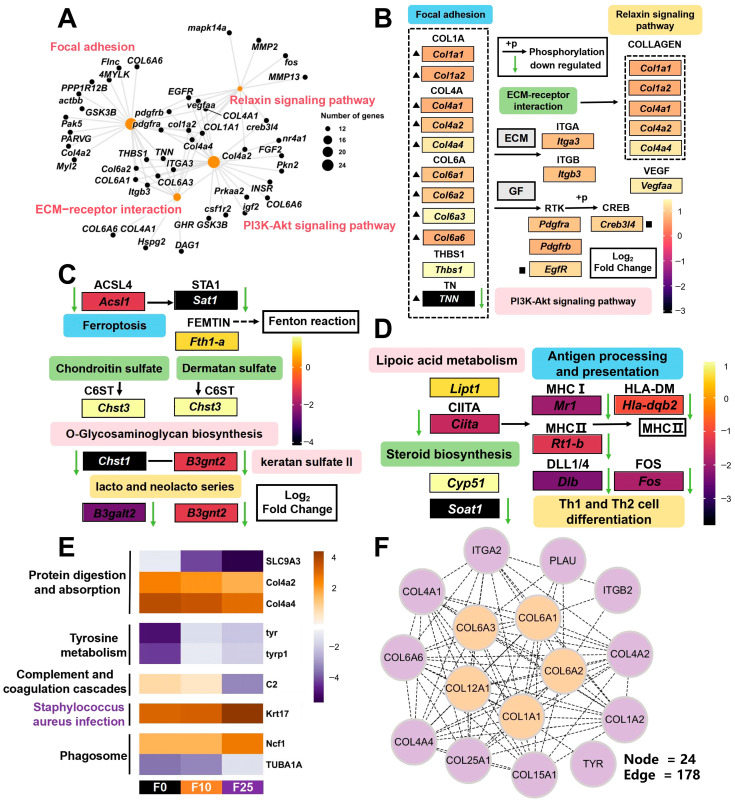
Identification of key signaling pathways and candidate genes under carbonate alkalinity exposure in the ovary. (**A**) Gene–pathway enrichment network maps constructed by targeting Focal adhesion, ECM–receptor interaction, PI3K-Akt signaling pathway, and Relaxin signaling pathway pathways in the F0 vs. F10 group; (**B**–**D**) Network regulation maps of key candidate genes and pathway constructs in the three comparison groups of F0 vs. F10, F0 vs. F25, and F10 vs. F25; (**E**) Expression heatmap of common DEGs in shared–enriched pathways; (**F**) Protein–protein interaction (PPI) network maps constructed based on the candidate gene sets. Green arrows indicated the downregulation of gene expression, all heatmaps with log2 Fold change (FC) value as legend, hub genes were highlighted in orange. (For interpretation of the references to colour in this figure legend, the reader is referred to the web version of this article.)

To further screen the key candidate genes in the significantly enriched pathways, since the F0 vs. F10 comparison group was enriched in more DEGs, the gene–pathway enrichment network was mapped by targeting the four significantly enriched signaling pathways (Figure 6A), in which genes *col1a1a*, *col1a2*, *col4a2*, and *col4a4* were common to all four pathways, and the differential gene expression was mapped based on the log2 (Fold change) value (Figure 5B). Focal adhesion was closely connected with the ECM–receptor interaction pathway and articulated with the PI3K-Akt signaling pathway and Relaxin signaling pathway, of which the PI3K-Akt signaling pathway and Relaxin signaling pathway were closely connected. Genes related to extracellular matrix (ECM) were significantly enriched and upregulated in all four significantly enriched signaling pathways. Similarly, mapping the differential gene regulatory networks of comparison groups F0 vs. F25 and F10 vs. F25, respectively (Figure 6C,D), revealed *sat1* of the Ferroptosis pathway was significantly downregulated, and it indirectly mediated the Fenton response in the F0 vs. F25 group. The genes *chst1*, *b3gnt2*, and *b3galt2*, which are related to glycan biosynthesis and metabolism, were also significantly downregulated; in the F10 vs. F25 group, genes related to Steroid biosynthesis and Lipoic acid metabolism, namely *ciita*, *mr1*, *hla-dqb2*, *rt1-b*, *soat1*, *dlb*, and *fos*, were all significantly downregulated.

Figure 6E compares the trends of expression changes in common genes in the common enrichment pathway in the three comparison groups of females, in which the expression of ovarian metabolism and the immunity-related genes *slc9a3*, *col4a2*, *col4a4*, and *C2* were downregulated with increasing alkalinity, and the genes related to autophagy and inflammatory response, namely *ncf1*, *tuba1a*, and *krt17*, were upregulated. Finally, the selected candidate genes were generated into gene sets, and gene interactions were determined and key genes for functional regulation were identified from a PPI network. A total of 24 Nodes and 178 Edges were generated, among which *col1a1*, *col6a1*, *col6a2*, *col6a3*, and *col12a1* were identified as hub genes (Figure 6F).

### 3.7. Identification and Functional Enrichment Analysis of DEGs in Testis

**Figure 7:**
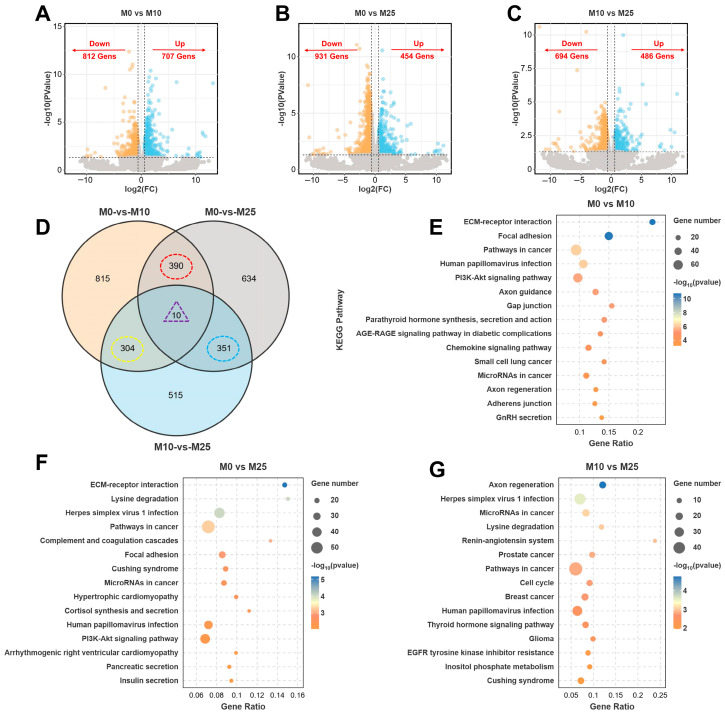
Transcriptome analysis of largemouth bass testis after carbonate alkalinity exposure. (**A**–**C**) Volcano plot statistics of upregulated and downregulated DEGs in the three comparison groups of M0 vs. M10, M0 vs. M25, and M10 vs. M25; (**D**) Differential Wayne’s plot statistics of the three comparison groups, with common DEGs circled in red, yellow, and blue, and common to all three groups circled in purple. (**E**–**G**) Statistics of KEGG pathways ranked in the top 15 (*p* < 0.05) for DEGs enrichment in the M0 vs. M10, M0 vs. M25, and M10 vs. M25 comparison groups.

For the three comparative groups of males (i.e., M0 vs. M10, M0 vs. M25, and M10 vs. M25), 1519 DEGs (707 upregulated, 812 downregulated), 1385 DEGs (454 upregulated, 931 downregulated), and 1180 DEGs (486 upregulated, 694 downregulated) were screened for, respectively (Figure 7A–C). More DEGs were identified in males than in females, and downregulated genes were more predominant. Differential Wayne plots of overlapping DEGs among three comparison groups of males (i.e., M0 vs. M10/M0 vs. M25, M0 vs. M10/M10 vs. M25, and M0 vs. M25/M10 vs. M25) (Figure 7D) identified a total of 390, 304, and 351 common DEGs, respectively, and 10 co-genes were also identified (Figure 7D). These common DEGs showed a more-pronounced cluster pattern than seen for females (Figure S3A–D), indicating that testis development was more affected than ovary development under alkaline conditions. Among the top 15 GO terms in each of the three comparison groups of male fish, the M0 vs. M10 group was more enriched in Cellular Component (CC) terms, such as cytoskeleton and cell projection; DEGs were more enriched in BP-related terms in the M0 vs. M25 group. In the M0 vs. M25 group, DEGs were more enriched in BP-related terms, such as chromosome organization and DNA conformation change; in the M10 vs. M25 group, DEGs were also more enriched in cell-binding-related terms, such as binding and ion binding (Figure S2B).

**Figure 8:**
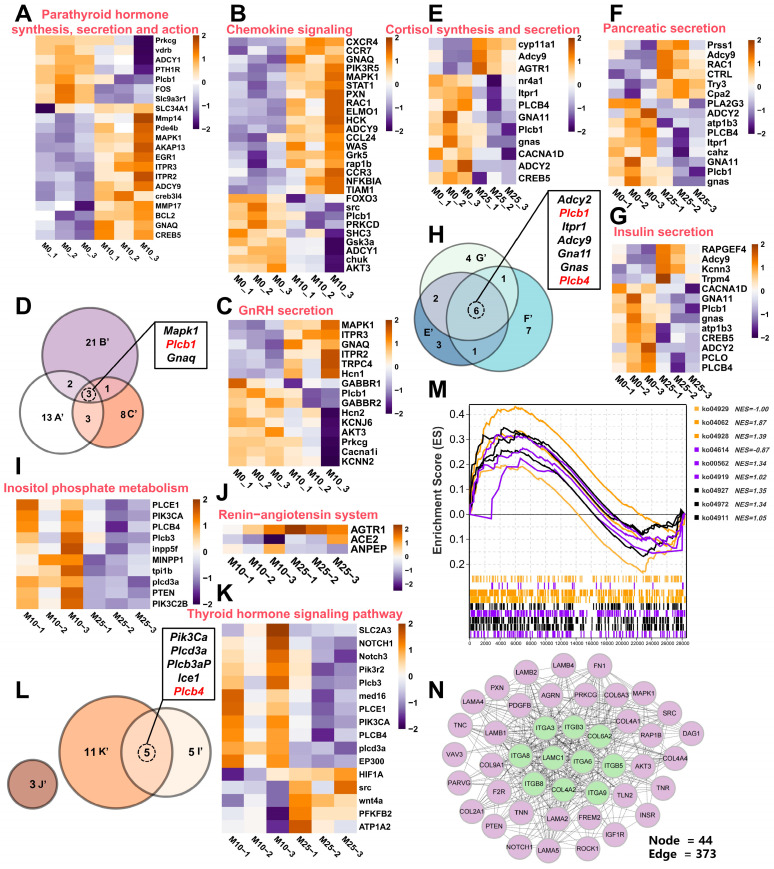
Identification of key signaling pathways and candidate genes in testis under carbonate alkalinity exposure. (**A**–**C**) Transcription level changes in key signaling pathways and DEGs in the M0 vs. M10 group; (**E**–**G**) Transcription level changes in key signaling pathways and DEGs in the M0 vs. M25 group; (**I**–**K**) Transcription level changes in key signaling pathways and DEGs in the M10 vs. M25 group; (**D**–**L**) Wayne diagram statistics of DEGs in key signaling pathways in the three comparative groups, with a total of genes in the Wayne diagrams marked in red; (**M**) Gene Set Enrichment Analysis (GSEA) analysis of nine signaling pathways; (**N**) PPI network diagram constructed based on the set of candidate genes. All heatmaps are illustrated with log2FC values and hub genes are highlighted in green.

The results of KEGG enrichment of DEGs in testis under carbonate alkalinity exposure found that DEGs were enriched in more signaling pathways in testis than in ovary, with the three comparison groups of M0 vs. M10, M0 vs. M25, and M10 vs. M25 being significantly enriched in 88, 34, and 34 signaling pathways, respectively (*p* < 0.05). Figure 7E–G show the top 15 signaling pathways of the three comparison groups of males. In the M0 vs. M10 group, three pathways related to the endocrine and immune system (Parathyroid hormone synthesis, secretion, and action; GnRH secretion; and chemokine signaling) were significantly enriched. Next, the three signaling pathways in the DEGs were analyzed by heatmap enrichment. The expression of genes related to thyroid hormone synthesis and inflammatory response were upregulated, such as *mapk1*, *blc2*, *itpr2*, *rac1*, *was*, and *hck*, as well as the expression of genes related to gonadal hormone synthesis, namely *mapk1*, *itpr3*, *itpr2*, and *gnaq* (Figure 8A–C). In addition, three genes in common (*mapk1*, *plcb1*, and *gnaq*) were identified in the three pathways (Figure 8D). In the M0 vs. M25 group, three pathways related to endocrine and digestive systems (Cortisol synthesis and secretion; pancreatic fluid secretion; Insulin secretion) were significantly enriched, wherein the genes related to Cortisol, Insulin, and pancreatic secretion were affected by carbonic acid base exposure and also showed different transcript-level changes (Figure 8E–G). Six genes in common, *adcy2*, *plcb1*, *itpr1*, *adcy9*, *gna11*, *gnas* and *plcb4*, were also identified in these three pathways (Figure 8H). In the M10 vs. M25 group, three signaling pathways related to endocrine and glucose metabolism (Inositol phosphate metabolism; Renin–angiotensin system; thyroid hormone) were significantly enriched. In these pathways, the DEGs involved in cell signaling were significantly downregulated; DEGs associated with thyroid hormone regulation were downregulated; gene *agtr1*, associated with the regulation of fluid homeostasis, was upregulated; and genes *ace2* and *anpep* were downregulated (Figure 8I–K). Five genes in common were identified in the thyroid hormone signaling pathway and Inositol phosphate metabolism pathway (Figure 8L). The GSEA scores showed the Renin–angiotensin system with GnRH secretion pathway with negative NES values, and the remaining seven pathways had positive NES values, indicating that carbonate alkalinity exposure induced the activation of signaling pathways related to testis development (Figure 8M). A PPI network map of candidate genes in testis was also constructed, and the top 10 (*igtb3*, *igtb5*, *igta8*, *igtb8*, *igta3*, *igta6*, *igta9*, *col4a2*, *col6a2*, and *lamc1*) were identified as hub genes (Figure 8F).

### 3.8. RT-qPCR Validation of DEGs in Relation to Indicators of Gonadal Developmental

**Figure 9:**
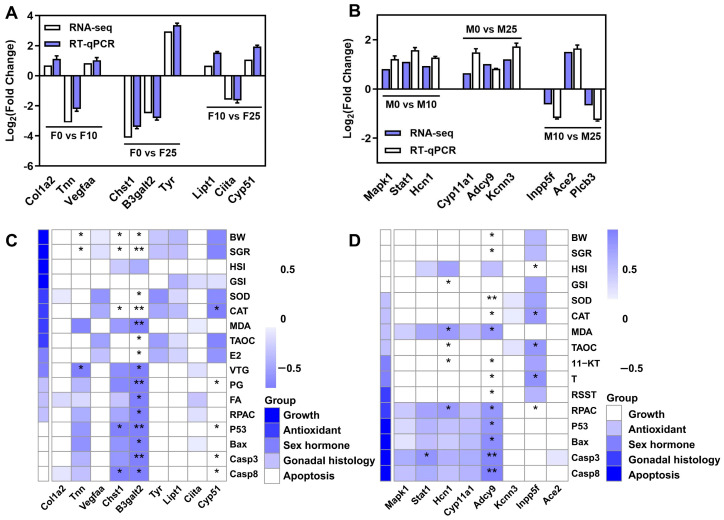
(**A**,**B**) Reverse Transcription-quantitative Polymerase Chain Reaction (RT-qPCR) validation of different comparison groups of largemouth bass ovaries and testes in response to carbonate alkalinity exposure; (**C**,**D**) Analysis of the correlation between nine key genes and gonadal developmental indices in ovaries and testes. *p* < 0.05 and *p* < 0.01 are indicated by “*” and “**”, respectively.

In the ovary, nine key genes related to signaling, cytogenesis, and immune regulation were selected from the three comparison groups of F0 vs. F10, F0 vs. F25, and F10 vs. F25; these were *Col1a2, Tnn, Vegfaa, Chst1, B3galt2, Tyr, Lipt1, Ciita,* and *Cyp51*. In the testis, nine key genes related to endocrine hormone synthesis and signal transduction were selected from the three groups of M0 vs. M10, M0 vs. M25, and M10 vs. M25; these were *Mapk1*, *Stat1*, *Hcn1*, *Cyp11a1*, *Adcy9*, *Kcnn3*, *Inpp5f*, *Ace2*, and *Plcb3*. RT-qPCR was performed to validate the RNA-seq sequencing results of these key genes in the ovary and testis of largemouth bass in response to carbonate alkalinity exposure. The RT-qPCR results were consistent with the transcriptome sequencing results (Figure 9A,B). Next, correlations between growth, antioxidant, sex hormones, gonadal histology, and apoptotic indices with key genes during male and female gonadal development after exposure to carbonate alkalinity was further analyzed. In the ovary, genes with significant changes in transcript levels affected by the exposure were more often positively correlated with these indices; in contrast, in the testis they were more often negatively correlated, with *B3galt2* and *Adcy9* showing significant changes in their correlation.

## 4. Discussion

### 4.1. Physiological Changes in Largemouth Black Bass in Response to Carbonate Alkalinity Exposure

Saline–alkaline aquaculture is a crucial strategy for sustainable aquaculture development, as it can alleviate the scarcity of freshwater resources and opens up new land for aquaculture. Currently, large-scale aquaculture in saline–alkaline water has been achieved with the mud crab *Scylla paramamosain*, Aral barbel *Luciobarbus brachycephalus*, and Pacific white shrimp *Litopenaeus vannamei* [9,35,36]. Previous studies have shown that a suitable alkalinity environment can promote physiological metabolism and increase energy metabolism, thereby potentially improving growth performance [37,38]. However, excessive carbonate alkalinity in aquatic environments can negatively impact organisms by disrupting osmotic pressure regulation and ammonia metabolism, ultimately inhibiting growth performance.

The largemouth bass has been shown to tolerate carbonate alkalinity up to 25 mmol/L; moreover, the nutritional quality of its muscle has been improved in alkaline environments of 15–20 mmol/L [39]. In this study, 80 days of carbonate alkalinity exposure resulted in a significant decrease in female body weight and SGR, while no significant changes were observed in males. The largemouth bass is a typical sexually dimorphic fish, and females tend to allocate more energy to ovary development prior to sexual maturity, and changes in lipid accumulation strategies under carbonate alkalinity exposure may have a greater impact on the female growth rate [32,40].

The liver is an important detoxification organ for steroid hormone metabolism, lipid metabolism, glucose metabolism, and other biological pathways in fish; thus, it plays a crucial role in responding to environmental stresses by regulating a variety of biological processes [41]. Previous studies have found that under acute carbonate stress, largemouth bass have significantly higher energy metabolism, requiring more energy to supply the individual [29]. Under chronic alkaline water exposure, common carp *Cyprinus carpio* experience oxidative stress and an enhanced glucose–lipid metabolism pathway to supply osmolarity-regulated organs [42]. The HSI represents the weight of the liver relative to the total body weight and is an important indicator of fish metabolism and health. In the previous study, it was found that as the carbonate alkalinity increased, the hepatocytes of largemouth bass swelled and the hepatic blood sinusoids continued to dilate [29]. In the long-term response to the high-alkalinity environment and the oxidative stress state, the largemouth bass needed to reserve more glycogen for energy allocation, which may contribute to the increase in HSI. Meanwhile, gonadal indices of both male and female fish decreased significantly after exposure to high alkalinity, suggesting that the alkaline environment inhibited reproductive function. When exposed to environmental stresses, largemouth bass may use more energy to maintain their physiological metabolic processes, thus reducing the energy required for reproductive development; this can include delayed gonadal development, a phenomenon that was observed in males and females of yellow catfish in response to a low-oxygen environment [21], and in male grass puffers *Takifugu alboplumbeus* in response to a low-temperature environment [43].

### 4.2. Carbonate Alkalinity Exposure Leads to Oxidative Stress

Numerous studies have shown that carbonate alkalinity exposure interferes with normal metabolic processes in fish and triggers the activation of antioxidant defense systems [11,13,44]. In this study, the SOD, CAT, and TAOC levels decreased significantly with increasing alkalinity in both females and males. SOD and CAT constitute the first line of antioxidant defense and are the core antioxidant enzymes for mitigating oxidative stress damage [13]. In both females and males of largemouth bass, SOD and CAT activities decreased dramatically at the alkalinity concentration of 25 mmol/L, indicating that the antioxidant enzyme system was damaged by chronic exposure to high alkalinity, resulting in excessive reactive oxygen species (ROS) accumulation that could not be scavenged in a timely manner, resulting in oxidative damage to the fish.

TAOC reflects the synergistic ability of antioxidant enzymes, like SOD, and the total antioxidant capacity of the body. The TAOC levels in the largemouth bass were low. MDA is a well-known marker of oxidative damage, specifically arising from lipid peroxidation; it is an end product of the process where ROS attack lipids, causing them to degrade [45]. In this study, the MDA content in both males and females increased significantly with exposure to higher alkalinity. This indicated that the exposure triggered MDA-mediated lipid peroxidation damage, which was accordingly associated with the decrease in the levels of SOD, CAT, and TAOC. Hence, the antioxidant defense system in the largemouth bass became imbalanced, leading to oxidative stress. This result is consistent with findings for common carp and Nile tilapia [42,46].

### 4.3. Carbonate Alkalinity Exposure Leads to Delayed Gonadal Development

Fish spermatogenesis and oogenesis are regulated by sex hormones. Together with the HPG axis, the sex hormones form a bi-directionally regulated endocrine network of reproduction, which precisely controls the development of the gonads, maturation of the gametes, and the reproductive behavior, a process that is particularly sensitive to environmental stresses [47,48]. For example, changes in certain environmental conditions, such as heavy metals, microplastics, sex hormones, dissolved oxygen, temperature, and ammonia nitrogen, can negatively impact reproductive processes and even lead to defects [49,50]. E2 is an essential sex hormone in ovary development, directly promotes oocyte maturation and follicular cell proliferation and induces the synthesis of VTG to supply oocytes with energy [21]. After carbonate alkalinity exposure, E2 levels in females were significantly reduced while VTG levels were significantly increased, with inconsistent changes in both. Although the exposure would have activated the HPG axis, the decrease in the gonadal indices explains the delayed ovarian development, resulting in a reduced capacity for ovarian E2 synthesis, as well as the redistribution of energy limiting the metabolic substrates required for gonadal steroid synthesis. Delayed ovarian development may have reduced the ability of oocytes to take up VTG, leading to its high accumulation in serum, a phenomenon observed in zebrafish *Danio rerio* [48] and rainbow trout *Oncorhynchus mykiss* [51]. 11-KT, a key androgen for the induction of sperm maturation, is synthesized mainly by testicular mesenchymal stromal cells in an enzymatic cascade reaction, which is catalyzed by the enzyme 11β-hydroxysteroid dehydrogenase (11β-HSD) to produce T. Testosterone is a prerequisite for 11-KT and is used to maintain spermatogonia proliferation [52]. In this study, the levels of T and 11-KT both decreased significantly after carbonate alkalinity exposure, and the changes were consistent. This suggests that carbonate alkalinity exposure similarly inhibited the HPG axis and disrupted the spermatogenesis process in the largemouth bass.

Largemouth bass is a typical non-synchronous spawning fish, meaning individuals can spawn several times during the breeding season. In the control group of females, most oocytes reached stage IV, but some were at stage II or III. In the control group of males, the testis had developed to period IV and was filled with a large number of secondary spermatogonia, spermatocytes, and spermatozoa. Salinity has been found to influence gamete quality in fish; for example, spotted scat had the highest gonadal index when reared at 25‰ [53].

In this study, it was demonstrated for the first time that carbonate alkalinity exposure retards gonadal development in largemouth bass, as reflected first by reduced gonadal indices in both females and males. Histological observations of ovary tissue found the proportions of PGs and FAs gradually increased with higher alkalinities, reaching 93.62% and 21.33%, respectively, in group CA25 females. In the testis, the incidence of spermatozoa in group CA25 was only 9.53%, significantly lower than in the control group males. Stress can lead to increased follicular atresia and a decreased percentage of spermatozoa, a process that can be mediated by the stress hormone cortisol [21,54,55]. Furthermore, elevated cortisol levels during sustained carbonate alkalinity exposure will inhibit the HPG axis, as was evidenced by the decreased levels of the sex hormones E2, 11-KT, and T.

TUNEL staining of gonadal tissues revealed that apoptotic signals were found in ovary and testis mesenchymal stromal cells, and the apoptosis-related genes *P53*, *Bax*, *Casp3*, and *Casp8* were upregulated in their expression. Apoptosis is a type of programmed cell death that is essential for maintaining the correct numbers of cells in a tissue and for eliminating cells that are harmful. Meanwhile, ultrastructural observations revealed that continuous exposure also led to a decrease in the number of mitochondria and blurring of cristae breaks. In fish, follicular atresia induces positive apoptotic signaling [56], while mitochondrial dysfunction is often associated with oxidative stress-induced ROS excess, triggered by mitochondrial autophagy [44]. Therefore, it is reasonable to hypothesize that excess ROS from oxidative stress-induced mitochondrial damage, leading to cellular ATP insufficiency and exacerbated apoptosis levels; at the same time, reduced sex hormone levels weakened the protective and proliferative regulation of mitochondria; together, this led to delayed gonadal development in the largemouth bass.

### 4.4. Molecular Mechanisms of Gonadal Developmental Regulation Under Carbonate Alkalinity Exposure

Under alkali exposure, a variety of physiological processes in aquatic organisms undergo adaptive changes, mainly involving the transport of substances across membranes, nutrient metabolism, and immune response [57]; there are few reports on the regulation of gonadal development. In females of largemouth bass, DEGs in the three comparison groups showed progressive molecular regulatory mechanisms. Pathways related to cell adhesion (Focal adhesion; ECM–receptor interaction) and signaling (PI3K-Akt signaling pathway; Relaxin signaling pathway) were significantly enriched in the F0 vs. F10 comparison group. In a previous study on largemouth bass gill tissues in response to acute high-alkalinity stress, Focal adhesion and ECM–receptor interaction were identified as key signaling pathways, and dysregulation of their interaction induced gill tissue lesions [29]. At the same time, low-alkalinity stress also activated the PI3K-Akt and Relaxin signaling pathways. Chinese mitten crab inhibited oxidative damage in gill tissues by upregulation of the Relaxin signaling pathway in response to high-temperature stress [58]; striped catfish *Pangasianodon hypophthalmus* was also activated by cold stress, which played a role in promoting cell growth and inhibiting apoptosis [59], whereas collagen, encoded by the core genes *col1a1a*, *col1a2*, *col4a2*, and *col4a4*, is a core component of the ECM [60], and upregulation of the expression of these genes suggests cell adhesion–signaling interactions to maintain follicular structural integrity. The glycosphingolipid biosynthesis and Ferroptosis pathways were significantly enriched in the F0 vs. F25 group. The simultaneous downregulation of the key genes *chst1*, *b3gnt2*, and *b3galt2* suggests that glycosphingolipid-mediated cellular recognition and signaling was disrupted, which may have led to the disruption of cellular communication during follicular development, thus affecting normal follicular development. Notably, the expression of the key gene for iron death, sat1, was significantly downregulated, suggesting that the abnormal mitochondrial morphology may be associated with SAT1-mediated inhibition of iron death. The downregulation of *sat1*, which encodes spermine/spermine acetyltransferase, leads to the accumulation of polyamines, which in turn promotes lipid peroxidation through the Fenton reaction and plays an important role in coping with oxidative damage [61,62]. Similar ovary regulatory mechanisms were also revealed by heatmaps of co-expressed gene and by the PPI networks; accompanied by elevated alkalinity, the downregulation of collagen gene expression, such as genes *col4a2* and *col4a4*, and upregulation of autophagy and inflammation-related genes, such as *ncf1*, *tuba1a*, and *krt17*, suggest that the activation of the collagen network at low alkalinity plays a protective role for the maintenance of ovary structure. In contrast, at high alkalinity, oxidative stress leads to increased ECM degradation, which ultimately led to increased follicular atresia and delayed ovarian development in the fish by inducing mitochondrial autophagy and inflammatory responses.

Compared to females, male testes expressed a higher molecular response in response to carbonate alkalinity exposure, with more DEGs identified. In the M0 vs. M10 comparison group, pathways such as GnRH secretion and chemokine signaling were significantly enriched, suggesting that carbonate alkalinity exposure activated the upstream regulation of the HPG axis. The upregulation of chemokine signaling pathway indicated that immune response-related genes, such as was and hck, synergistically acted in concert to scavenge early oxidative damage [63]. Cortisol synthesis and secretion were significantly enriched in the M0 vs. M25 comparison group. In response to environmental stress, cortisol, as a major stress hormone involved in the regulation of numerous physiological processes, including energy metabolism, ionic regulation, and osmotic homeostasis [64,65], coordinated the overall energy allocation by inhibiting the HPG axis, which was demonstrated by a decrease in the levels of 11-KT and T levels in male largemouth bass. Insulin secretion and pancreatic fluid secretion are both important endocrine regulatory systems, and the synchronized dysregulation in response to high-alkalinity exposure responds to the restricted energy requirements for spermatogenesis, and the disturbed synthesis of digestive enzymes indirectly contributes to the nutrient supply for testicular development [66]. This was evidenced by the fact that only 9.53% spermatogenesis was observed in group CA25. More DGEs associated with thyroid hormone regulation were significantly downregulated in the M10 vs. M25 comparison group. The thyroid in mammals primarily regulates metabolic activities, but in fish it can be directly involved in gonadal development regulation [67]. Similarly to females, the lack of energy required for testicular development under high-alkalinity stress also resulted in defective testicular development. The overall results show that the testis of the largemouth bass was affected by oxidative stress and endocrine disruption, which negatively impacted testis development, which could delay maturation.

## 5. Conclusions

This study investigated the impact of chronic carbonate alkalinity exposure on largemouth bass gonadal development. Alkaline water altered energy allocation, exerting a greater influence on the growth rate of females. Prolonged exposure disrupted the antioxidant system, causing excess ROS which damaged mitochondria and increased apoptosis. Carbonate also inhibited the HPG axis, dysregulating reproductive endocrinology and reducing the levels of E2, 11-KT, and T sex hormones. Phenotypically, this manifested as a lower GSI, increased the ratio of primary oocytes to follicular atresia, and decreased spermatogenesis proportions, and caused more severe effects on gonadal development overall as a result of alkalinity exposure. Transcriptomics revealed that chronically low-alkalinity activated collagen networks and cellular adhesion, maintaining gonad structure. Conversely, chronically high-alkalinity-induced oxidative stress, disrupting energy metabolism and the endocrine system, leading to tissue damage and delayed development. In the subsequent process of saline–alkaline water culture of largemouth bass, the monitoring and management of carbonate alkalinity of the water should be strengthened, alkalinity reduction techniques should be developed, and feed additives that can effectively alleviate oxidative stress damage should be combined, thus enhancing the reproductive performance of largemouth bass. In general, these findings provide insights into the physiological response of largemouth bass to alkaline environments and the associated reproductive disorders.

## Data Availability

All data generated or analyzed during this study are included in this published article and its Supplementary Materials information files.

## Supplementary Materials

The following supporting information can be downloaded at: https://www.mdpi.com/article/10.3390/antiox14091042/s1. Figure S1: (A) Upset plot of DEGs in the female comparison group; (B-D) Heatmap of expression clustering of common DEGs in the three comparison groups. Figure S2: (A) Statistics of GO terms enriched for DEGs in the three comparison groups F0 vs F10, F0 vs F25 and F10 vs F25; (B) Statistics of GO terms enriched for DEGs in the three comparison groups M0 vs M10, M0 vs M25 and M10 vs M25. Figure S3: (A) Upset plot of DEGs in the male comparison group; (B-D) Heatmap of expression clustering of common DEGs in the three comparison groups. Table S1. Statistics on sample numbers used in the experimental analysis. Table S2. Primers used for RT-qPCR in this study. Table S3. Sequencing data statistics for 18 transcriptome samples.
