# Supplementary material for: Chronic Carbonate Alkalinity Exposure Induces Dysfunction in Ovary and Testis Development in Largemouth Bass Micropterus salmoides by Oxidative Damage and Sex-Specific Pathways
Source: Antioxidants (Basel). 2025 Aug 23;14(9):1042. doi: 10.3390/antiox14091042 (PMC12466519; doi:10.3390/antiox14091042)
Supplement: Supplementary file 1 [file antioxidants-14-01042-s001.zip › antioxidants-3802332-supplementary-figures.pdf]

## Supplemental Figures

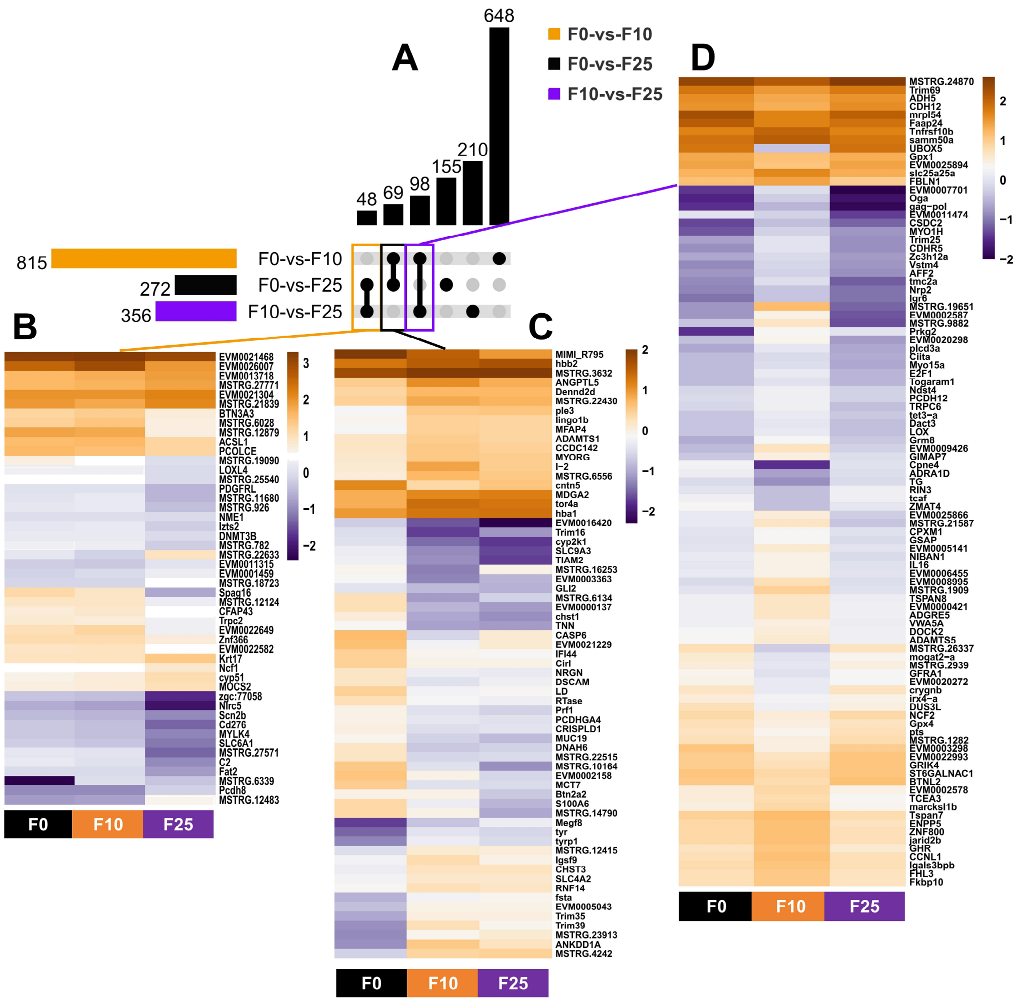


**Figure S1.** (A) Upset plot of DEGs in the female comparison group; (B-D) Heatmap of expression clustering of common DEGs in the three comparison groups.

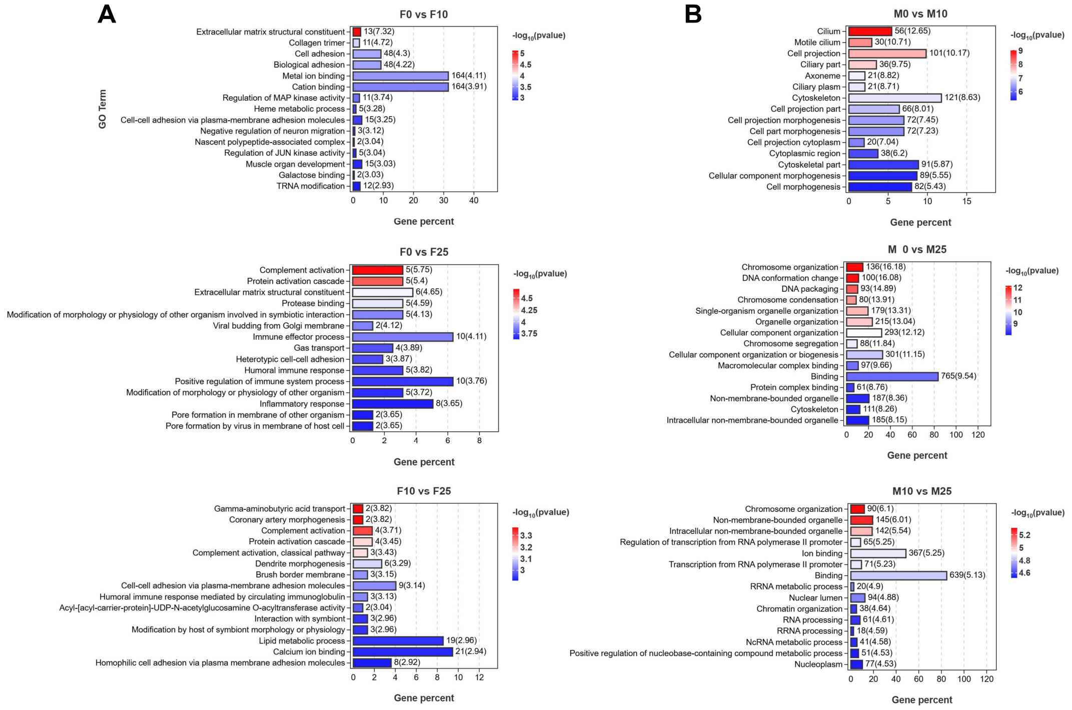


**Figure S2.** (A) Statistics of GO terms enriched for DEGs in the three comparison groups F0 vs F10, F0 vs F25 and F10 vs F25; (B) Statistics of GO terms enriched for DEGs in the three comparison groups M0 vs M10, M0 vs M25 and M10 vs M25.

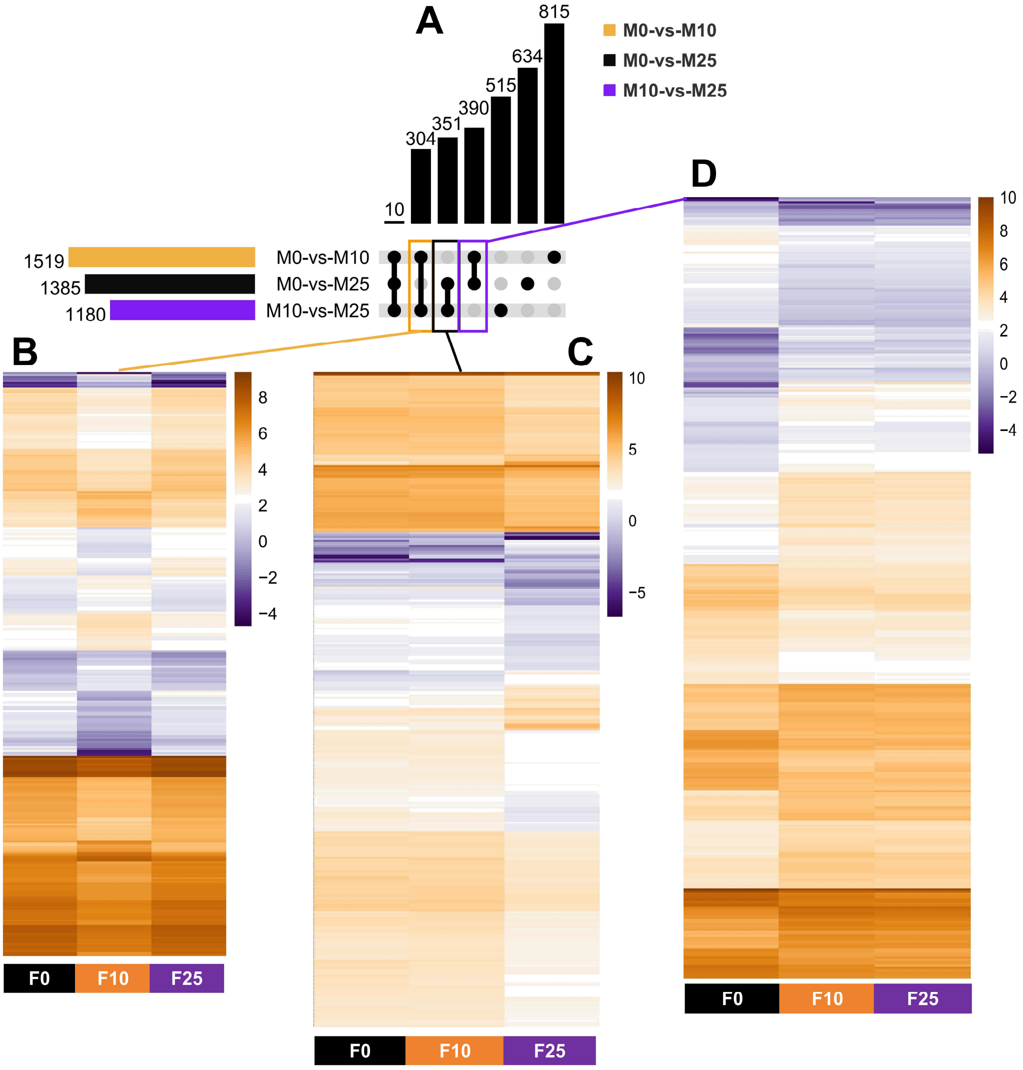


**Figure S3.** (A) Upset plot of DEGs in the male comparison group; (B-D) Heatmap of expression clustering of common DEGs in the three comparison groups.
