# Supplementary material for: Chronic Carbonate Alkalinity Exposure Induces Dysfunction in Ovary and Testis Development in Largemouth Bass Micropterus salmoides by Oxidative Damage and Sex-Specific Pathways
Source: Antioxidants (Basel). 2025 Aug 23;14(9):1042. doi: 10.3390/antiox14091042 (PMC12466519; doi:10.3390/antiox14091042)
Supplement: Supplementary file 1 [file antioxidants-14-01042-s001.zip › antioxidants-3802332-supplementary-tables.pdf]

Supplemental Tables

Table S1. Statistics on sample numbers used in the experimental analysis.

Experimental analyses	F0	F10	F25	M0	M10	M25
Growth performance	n=15	n=15	n=15	n=15	n=15	n=15
Antioxidant indicators	n=9	n=9	n=9	n=9	n=9	n=9
Sex hormone measurements	n=9	n=9	n=9	n=9	n=9	n=9
HE staining	n=6	n=6	n=6	n=6	n=6	n=6
TUNEL staining	n=6	n=6	n=6	n=6	n=6	n=6
TEM analysis	n=2	n=2	n=2	n=2	n=2	n=2
RNA-seq	n=9	n=9	n=9	n=9	n=9	n=9

Table S2. Primers used for RT-qPCR in this study.

Gene	Primer sequence (5′-3′)	TM (°C)	Amplicon size (bp)	Amplification efficiency (%)	Gene ID
Col1a2	F:TGCCACCATCAAGTCGCTCAAC<br>R:TTGCCGCTCTCGCCATCCAT	59.8/60.3	447	103.6	119906985
Tnn	F:TGGCAATAAGAGGAGCGGTAT<br>R:TCAGTAGGAGCAGGAGTGTTG	54.8/55	246	98.0	119902121
Vegfaa	F:GCGGCTCTTCACTTATCAACT<br>R:CACACTCCATTGCTTCGTCAT	54.3/54.9	235	101.7	119896891
Chst1	F:TTACGCCTGCTGTGCTCTG<br>R:GACCTCTGGATGCTGATTGAAC	55.8/55.2	261	103.5	119893050
B3galt2	F:GGAGAAGCCAACCAGAGCAATT<br>R:GCGATGAGGAGGACGAGGAA	57.2/57.0	305	96.5	119900310
Tyr	F:CCACCAGCCTCTCAAGACAAG<br>R:CAATAAGCAGCACAGCAACGA	56.5/55.8	284	102.8	119883400
Liptl1	F:TGGCTCTGGAGGACTGGAT<br>R:TGGCGTTGCTCTGGATACC	54.8/55.1	280	101.5	119908658
Ciita	F:CGATGTTGCTGCCTCTGGTTCC<br>R:TCCGCTTGTGCCTCTGTGGTT	60.1/60.4	272	97.6	119885938
Cyp51	F:GGTGGAGAGGATGGAGTTCAA<br>R:GGTGTGGATCATTGTGGTGTAG	54.9/55.1	226	102.2	119907680
Mapk1	F:GATGGTGTGCTCTGCCTACGA<br>R:AATGGTTGGTGTGCGGATGATG	58.0/57.9	175	98.5	119891295
Stat1	F:TGGAAGCAGCGGCAGCAGAT<br>R:TGACGACGACAGGCAGAGACAT	60.5/59.8	252	96.4	119900506
Hcn1	F:CATTGACCGATTGGACCGCATT<br>R:GCCTGACTGAAGAGCCTGTGT	58.0/58.2	266	98.7	119915098

Cyp11a1	F:TTGCTGGATGAAGTTGGTGAG<br>R:ACTGCCTGTAGATGTTATGGATG	54.4/54.3	355	104.4	119894014
Adcy9	F:CCACCAACCACCAGACAGGAT<br>R:GAGGAACCAGACCAGGAGAAGG	57.8/57.8	171	95.2	119899433
Kcnn3	F:GCATCGCTATGACTATGGAGAG<br>R:GAGGAGGCATCGGTGAAGA	54.4/54.1	238	102.2	119897575
Inpp5f	F:GGAGACACCATCAGCAGACAGT<br>R:TTAGACAGGAGCAGCAGCACAT	58.1/58	313	100.5	119917416
Ace2	F:TGAACCTCCTGCCTGCTGACTT<br>R:AACACCTGCCACCTCCATTCCT	59.9/59.9	130	100.3	119890262
Plcb3	F:ACTGGCTGTCGCTGTGTAGAG<br>R:CGGTGGTGTTGGTGCTTCTTC	58.0/58.0	359	105.5	119911817
P53	F:AGATTGAATGGTGGTGGG<br>R:GTTCTGGCGGACTGGA	58.0/58.0		[33]	
Bax	F:TTCTACTTTGCGTGTCGGCT<br>R:AACAGTCAGCGTTCCATCCA	58.0/58.0		[33]	
Casp3	F:AGCATCCACCCAACTATCCT<br>R:ACGTGCCCCAATTAAAAGCA	58.0/58.0		[33]	
Casp8	F:GAGACAGACAGCAGACAACCA<br>R:TTCCATTTCAGCAAACACATC	56.0/56.0		[22]	
β-actin	F:ATCGCCGCACTGGTTGTTGAC<br>R:CCTGTTGGCTTTGGGGTTC	54.9/53.8		[29]	

Table S3. Sequencing data statistics for 18 transcriptome samples.

Sample	Raw reads	Raw Bases	Clean reads (%)	Clean Bases	Q20 (%)	Q30 (%)	GC(%)	Mapped (%)
F0_1	40504744	6.08 G	40339996 (99.59%)	6.01 G	98.32	95.21	49.48	96.16
F0_2	54523854	8.18 G	54349186 (99.68%)	8.10 G	98.23	95.07	49.54	96.74
F0_3	54171692	8.13 G	54014216 (99.71%)	8.05 G	98.29	95.19	49.7	96.88
F10_1	48930976	7.34 G	48792508 (99.72%)	7.27 G	98.24	95.06	49.38	96.12
F10_2	47699798	7.15 G	47565458 (99.72%)	7.09 G	98.27	95.18	49.43	96.07
F10_3	47215978	7.08 G	47079160 (99.71%)	7.01 G	98.17	94.93	49.70	96.53
F25_1	48984974	7.35 G	48831186 (99.69%)	7.28 G	98.18	94.95	49.43	96.18
F25_2	55222884	8.28 G	55083950 (99.75%)	8.20 G	98.38	95.38	49.34	96.77
F25_3	54200198	8.13 G	54023476 (99.67%)	8.05 G	98.21	95.05	50.91	96.90
Average	50161678	7.52 G	50008793 (99.69%)	7.45 G	98.25	95.11	49.66	96.48
M0_1	48009938	7.20 G	47856054 (99.68%)	7.15 G	98.48	95.47	49.02	93.67
M0_2	43675498	6.55 G	43534726 (99.68%)	6.50 G	98.47	95.45	48.91	94.43
M0_3	41793546	6.27 G	41662516 (99.69%)	6.23 G	98.51	95.58	49.14	94.08
M10_1	39063156	5.86 G	38964714 (99.75%)	5.82 G	98.34	95.05	49.22	94.31
M10_2	43017008	6.45 G	42868668 (99.66%)	6.41 G	98.30	95.07	49.28	94.08

M10_3	43761286	6.56 G	43632170 (99.70%)	6.52 G	98.33	95.13	48.95	93.75
M25_1	44033354	6.61 G	43897812 (99.69%)	6.56 G	98.50	95.49	49.24	94.36
M25_2	42574496	6.39 G	42446212 (99.70%)	6.35 G	98.46	95.39	49.29	94.38
M25_3	37619446	5.64 G	37487744 (99.65%)	5.60 G	98.48	95.47	49.38	94.33
Average	426164148	6.39 G	42483402 (99.69)	6.35 G	98.43	95.34	49.16	94.15
